# Birt-Hogg-Dubé syndrome initially diagnosed as tuberous sclerosis complex

**DOI:** 10.1016/j.jdcr.2019.02.009

**Published:** 2019-04-05

**Authors:** Deeti J. Pithadia, Alison M. Treichel, Chyi-Chia Richard Lee, Edward W. Cowen, W. Marston Linehan, Joel Moss, Thomas N. Darling

**Affiliations:** a Department of Dermatology, Uniformed Services University of the Health Sciences, Bethesda, Maryland; b Pulmonary Branch, National Heart, Lung, and Blood Institute, National Institutes of Health, Bethesda, Maryland; c Laboratory of Pathology, Center for Cancer Research, National Cancer Institute, National Institutes of Health, Bethesda, Maryland; d Dermatology Branch, National Institute of Arthritis and Musculoskeletal and Skin Diseases, National Institutes of Health, Bethesda, Maryland; e Urologic Oncology Branch, Center for Cancer Research, National Cancer Institute, National Institutes of Health, Bethesda, Maryland

**Keywords:** angiofibroma, Birt-Hogg-Dubé syndrome, fibrofolliculoma, trichodiscoma, tuberous sclerosis complex, AF, angiofibroma, BHDS, Birt-Hogg-Dubé syndrome, FF, fibrofolliculoma, mTORC1, mechanistic target of rapamycin complex 1, TD, trichodiscoma, TSC, tuberous sclerosis complex

## Introduction

Birt-Hogg-Dubé syndrome (BHDS) is an autosomal dominant disorder caused by a pathogenic variant of the *FLCN* gene. Tuberous sclerosis complex (TSC) results from a heritable pathogenic variant of *TSC1* or *TSC2*. Both BHDS and TSC may present with papules on the nose and cheeks; fibrofolliculomas (FFs) and trichodiscomas (TDs) are observed in BHDS, and multiple angiofibromas (AFs) are classically seen in TSC. In both disorders, skin lesions may appear in association with bilateral pulmonary cysts and renal tumors. Similarities in the most striking features of BHDS and TSC may render it challenging to distinguish the 2 disorders.

## Case report

**Fig 1:**
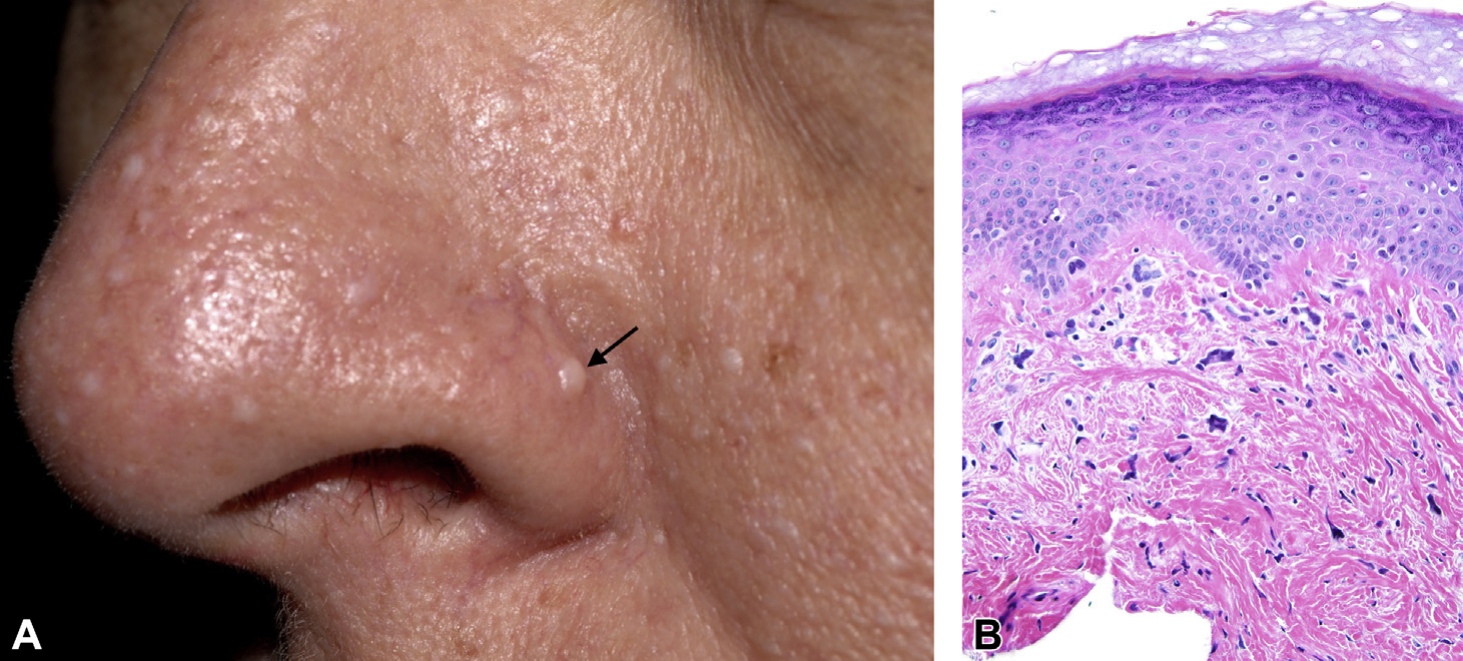
Gross and microscopic appearances of papules on the patient's face. **A**, Multiple gray-white to skin-colored papules visible on the nose, chin, and medial aspects of the cheeks of our patient during her initial visit. A shave biopsy specimen of 1 of the papules (*arrow*) was obtained at this visit. **B**, Photomicrograph of a hematoxylin–eosin-stained section of the biopsy specimen reveals stellate-shaped dermal fibroblasts surrounded by coarse collagen, which is characteristic of angiofibroma.

A 45-year-old woman presented for dermatologic examination after a clinical diagnosis of TSC based on a history of facial papules since she was in her twenties, 1 of which was a biopsy-proven AF, as well as seizures during her teens, multiple pneumothoraces, and renal cysts. The physical examination revealed numerous gray-white to skin-colored papules on the nose and medial aspects of her cheeks (Fig 1, *A*) and a single gingival papule, but no other significant mucocutaneous findings. A shave biopsy specimen of a nasal alar papule was obtained, and it revealed stellate-shaped fibroblasts with fibrotic dermal collagen displacing solar elastosis, which is characteristic of AF (Fig 1, *B*).^1^ Computed tomography scans of her chest, abdomen, and pelvis revealed multiple pulmonary and renal cysts. A magnetic resonance imaging scan of her brain revealed no TSC-associated changes.

**Fig 2:**
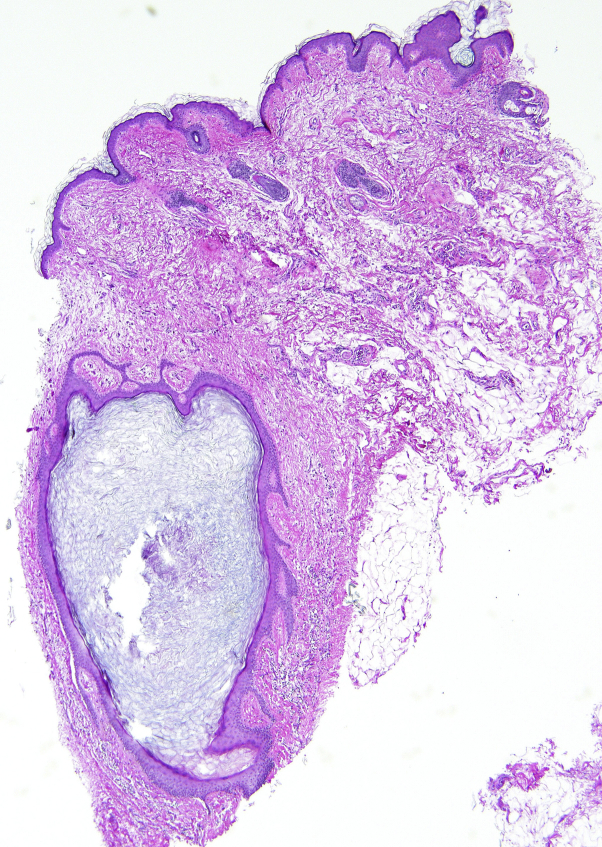
Microscopic appearance of a papule on the posterior aspect of the patient's sister's neck. Photomicrograph of a hematoxylin–eosin-stained section of a papule excised from the posterior aspect of the patient's sister's neck using punch biopsy. Histopathology reveals a cystically dilated hair follicle containing keratinous debris and surrounded by prominent mantle-like epithelium in a fenestrated pattern, which is characteristic of fibrofolliculoma. The collagen directly surrounding this hair follicle does not display concentric arrangement that is more typical of angiofibroma.

The lack of additional TSC-related mucocutaneous and internal findings introduced uncertainty in the diagnosis of TSC and prompted evaluation of the patient's 56-year-old sister. Her sister had no significant pulmonary, renal, or neurologic history. Computed tomography scans of her chest, abdomen, and pelvis revealed pulmonary and renal cysts, and a magnetic resonance imaging scan of her brain showed no significant abnormalities. Her skin examination revealed gray-white papules on the face and neck, gingival papules, and axillary and inframammary acrochordons, all characteristics of BHDS. Punch biopsy specimens obtained from papules on her neck and posterior ear revealed cystically dilated infundibular portion of hair follicles containing keratin debris with epithelial strands emanating from the follicular infundibulum (Fig 2) with characteristic dermal collagen, all of which are representative features of FF.^1^, ^2^

Given her sister's findings, the patient was reevaluated for additional skin lesions consistent with BHDS. Punch biopsy specimens were obtained from 2 papules on her jawline, and the histopathologic examination showed findings of FF. Germline *FLCN* testing revealed a nonsense variant (c.1844C>G, p.Tyr463X) in exon 12.

## Discussion

The key clinical features of BHDS may mimic those observed in patients with TSC, and this may generate diagnostic confusion. AFs in TSC generally appear in childhood as pink-red, telangiectatic papules on the cheeks and nose. FFs and TDs in BHDS typically manifest during adulthood as gray-white papules with occasional follicular dells on the face, neck, and behind the ears.^2^ However, TSC-associated AFs occasionally manifest later in life, and some patients with BHDS may develop AFs. A patient with genetically proven BHDS was reported in whom 39 of 41 papules removed by shave excision were AFs while just 2 were FFs.^3^ A family study of patients with BHDS found that 10 of 51 families had ≥1 member with histologically proven AF.^4^ Therefore, BHDS may still be considered in patients with AFs, particularly if AFs have delayed onset and limited erythema, as seen in our patient. In addition, BHDS may present with cystic lung disease and spontaneous pneumothorax, similar to TSC-associated pulmonary lymphangioleiomyomatosis. Both disorders are also associated with benign renal cysts, and BHDS patients can develop renal cancers that may appear similar on imaging to renal angiomyolipomas, which are classic for TSC.

When evaluating a patient with features of both TSC and BHDS, a thorough dermatologic examination and medical workup that assesses for unique characteristics of each disorder must be performed. TSC-specific mucocutaneous findings include hypopigmented macules, shagreen patches, fibrous cephalic plaques, periungual fibromas, and dental pitting. TSC patients may also have cardiac rhabdomyomas, neurologic tumors, seizures, and neuropsychiatric disorders. BHDS patients generally lack the aforementioned findings. When clinical findings are nondiagnostic, skin biopsy specimens may be obtained from papules on the lateral aspect of the face or neck. Obtaining a punch biopsy specimen with vertical sectioning of these papules is preferred over shave biopsy specimens with transverse sectioning. Deeper tissue samples are more likely to reveal an intact follicular structure and epithelial strands that are diagnostic of FF. Evaluation of relatives and genetic testing may also aid in the diagnosis.

In rare cases, dermatologic features that are considered distinctive for BHDS may occur in patients with TSC, and vice versa. FFs^5^ and multiple acrochordons^6^ are classic for patients with BHDS but have been reported in patients with TSC. Likewise, periungual fibromas^7^ and intraoral papules^8^ are classic for TSC but have been observed in patients with BHDS. The histopathologic appearances of AFs and FFs are another point of potential overlap between BHDS and TSC. In some FFs, AF-like stellate fibroblasts are present and the epithelial streamers surrounding hair follicles may be subtle. AFs may exhibit sebaceous components within hair follicles, mimicking FFs.^1^ In cases where detailed gross and histologic evaluation of the skin accompanied by medical workup are inconclusive, genetic testing may be crucial to distinguish the disorders.

**Fig 3:**
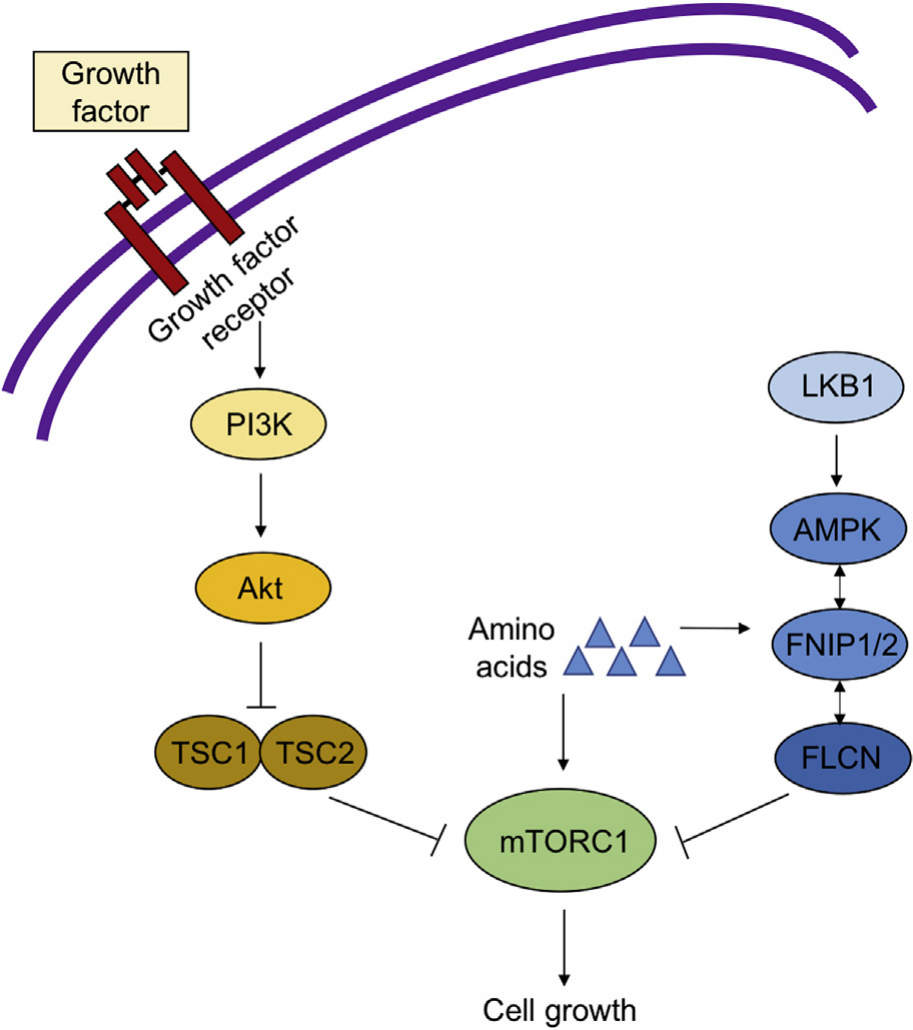
Aberrant mechanistic target of rapamycin complex 1 (mTORC1) signaling in tuberous sclerosis complex (TSC) and Birt-Hogg-Dubé syndrome. Activation of PI3K-Akt by extracellular growth factors results in inhibition of the TSC1–TSC2 protein complex, which leads to activation of mTORC1. Pathogenic variants in *TSC1* or *TSC2* inactivate this complex and result in mTORC1 activation. FLCN protein is activated by complex formation with FNIP1/2 and AMPK secondary to signals from LKB1 and amino acids. FLCN may show excitatory or inhibitory effects on mTORC1, depending on the type of cell.

On a molecular level, both TSC and BHDS are caused by pathogenic gene variants that alter signaling through mechanistic target of rapamycin complex 1 (mTORC1). In TSC, loss of function of the TSC1–TSC2 complex causes activation of mTORC1, inducing cellular growth. The downstream effects resulting from abnormal FLCN are less clearly elucidated; *FLCN* mutations may result in either increased or decreased mTORC1 activity depending on cell type (Fig 3).^8^ It is possible that the loss of TSC1, TSC2, or FLCN function results in shared abnormalities in downstream signaling pathways. This may account for the similarities in systemic findings between the 2 disorders.

Differentiating TSC and BHDS is crucial for appropriate surveillance and treatment. BHDS is associated with a 12% to 34% lifetime risk of potentially life-threatening renal cancers, including hybrid, chromophobe, and, occasionally, clear cell carcinoma.^8^ This is compared with a 2% to 4% risk observed in patients with TSC.^9^ Therefore, screening with renal magnetic resonance imaging at least every 3 years—which is recommended for both patients with BHDS^8^ and patients with TSC^10^—is especially imperative in patients with BHDS. Systemic mechanistic target of rapamycin inhibitors have proven efficacy for TSC-associated internal tumors, and their utility for BHDS warrants additional study.^8^

This case report demonstrates potential challenges in clinically discerning TSC and BHDS and also emphasizes the utility of evaluation of relatives, punch biopsy specimens, and genetic testing in making the distinction.

## References

[1] Misago N., Kimura T., Narisawa Y. (2009). Fibrofolliculoma/trichodiscoma and fibrous papule (perifollicular fibroma/angiofibroma): a revaluation of the histopathological and immunohistochemical features. J Cutan Pathol.

[2] Aivaz O., Berkman S., Middelton L., Linehan W.M., DiGiovanna J.J., Cowen E.W. (2015). Comedonal and cystic fibrofolliculomas in Birt-Hogg-Dubé syndrome. JAMA Dermatol.

[3] Schaffer J.V., Gohara M.A., Mcniff J.M., Aasi S.Z., Dvoretzky I. (2005). Multiple facial angiofibromas: a cutaneous manifestation of Birt-Hogg-Dubé syndrome. J Am Acad Dermatol.

[4] Toro J.R., Wei M.H., Glenn G.M. (2008). BHD mutations, clinical and molecular genetic investigations of Birt-Hogg-Dubé syndrome: a new series of 50 families and a review of published reports. J Med Genet.

[5] Misago N., Narisawa Y. (2009). Fibrofolliculoma in a patient with tuberous sclerosis complex. Clin Exp Dermatol.

[6] Baykal C. (2018). Acrochordons on the neck; a remarkable clinical feature of tuberous sclerosis showing different patterns. J Eur Acad Dermatol Venereol.

[7] DiCicco B., Johnson W., Allred J., Soldano A.C., Ramsdell W.M. (2016). Koenen’s tumor and facial angiofibromas in a case of Birt-Hogg-Dubé syndrome: a cutaneous contribution to growing evidence of a relationship with tuberous sclerosis complex. JAAD Case Rep.

[8] Schmidt L.S., Linehan W.M. (2015). Clinical features, genetics and potential therapeutic approaches for Birt-Hogg-Dubé syndrome. Expert Opin Orphan Drugs.

[9] Yang P., Cornejo K.M., Sadow P.M. (2014). Renal cell carcinoma in tuberous sclerosis complex. Am J Surg Pathol.

[10] Krueger D.A., Northrup H. (2013). Tuberous sclerosis complex surveillance and management: recommendations of the 2012 International Tuberous Sclerosis Complex Consensus Conference. Pediatr Neurol.

